# Accurate inference of shoot biomass from high-throughput images of cereal plants

**DOI:** 10.1186/1746-4811-7-2

**Published:** 2011-02-01

**Authors:** Mahmood R Golzarian, Ross A Frick, Karthika Rajendran, Bettina Berger, Stuart Roy, Mark Tester, Desmond S Lun

**Affiliations:** 1 Phenomics and Bioinformatics Research Centre, Australian Centre for Plant Functional Genomics, School of Mathematics and Statistics, University of South Australia, Mawson Lakes, SA, 5095, Australia; 2 School of Mathematics and Statistics, University of South Australia, Mawson Lakes, SA, 5095, Australia; 3 Australian Centre for Plant Functional Genomics, University of Adelaide, Glen Osmond SA 5064, Australia; 4 Department of Computer Science and Center for Computational and Integrative Biology, Rutgers University, Camden, NJ 08102, USA

## Abstract

With the establishment of advanced technology facilities for high throughput plant phenotyping, the problem of estimating plant biomass of individual plants from their two dimensional images is becoming increasingly important. The approach predominantly cited in literature is to estimate the biomass of a plant as a linear function of the projected shoot area of plants in the images. However, the estimation error from this model, which is solely a function of projected shoot area, is large, prohibiting accurate estimation of the biomass of plants, particularly for the salt-stressed plants. In this paper, we propose a method based on plant specific weight for improving the accuracy of the linear model and reducing the estimation bias (the difference between actual shoot dry weight and the value of the shoot dry weight estimated with a predictive model). For the proposed method in this study, we modeled the plant shoot dry weight as a function of plant area and plant age. The data used for developing our model and comparing the results with the linear model were collected from a completely randomized block design experiment. A total of 320 plants from two bread wheat varieties were grown in a supported hydroponics system in a greenhouse. The plants were exposed to two levels of hydroponic salt treatments (NaCl at 0 and 100 mM) for 6 weeks. Five harvests were carried out. Each time 64 randomly selected plants were imaged and then harvested to measure the shoot fresh weight and shoot dry weight. The results of statistical analysis showed that with our proposed method, most of the observed variance can be explained, and moreover only a small difference between actual and estimated shoot dry weight was obtained. The low estimation bias indicates that our proposed method can be used to estimate biomass of individual plants regardless of what variety the plant is and what salt treatment has been applied. We validated this model on an independent set of barley data. The technique presented in this paper may extend to other plants and types of stresses.

## Introduction

Plant biomass is an important factor in the study of functional plant biology and growth analysis, and it is the basis for the calculation of net primary production and growth rate [1-4]. Depending on the available budget, accuracy required, structure and composition of the vegetation, and also different disciplines of plant biology, there are several techniques to measure plant biomass [5]. In the study of biomass of an individual plant, shoot dry weight is one of the acceptable measures. This method is typically used to estimate a plant's yield, but it is also an accurate measure of plant biomass.

The conventional means of determining shoot dry weight (SDW) is the measurement of oven-dried samples. In this method, tissue is harvested and dried, and then shoot dry weight is measured at the end of the experiment. To investigate the biomass of a large number of plants, this method is very time consuming and labor intensive. Also, since this method is destructive, it is impossible to take several measurements on the same plant at different time points. Therefore, an imaging method has been proposed to infer plant biomass accurately as a non-destructive and fast alternative. The Plant Accelerator [6] and the High Resolution Plant Phenotyping Centre [7] in Australia, the Leibniz Institute of Plant Genetics and Crop Plant Research (IPK) in Germany [8], the Institute of Biological, Environmental and Rural Sciences (IBERS) in the UK [9], and PHENOPSIS system being built by the National Institute for Agricultural Research (INRA) in Montpellier, France [10], have established or are planning to establish advanced plant phenotyping facilities that each provide the capability of hundreds to thousands of plants to be automatically imaged from standard positions and then analyzed via image analysis programs every day.

Digital image analysis has been an important tool in biological research and also has been applied to satellite images, aerial photographs and macroscopic and microscopic images [11]. A relevant application of image analysis which has been used for decades is in the area of remote sensing forestry and precision agriculture in which the area of plant species cover and the biomass of the above-ground canopy are estimated from satellite and airborne images [12-20]. These techniques have found a recent application in estimating the biomass of individual plants in a controlled environment and also in the field. There have been only a few projects on the application of image analysis techniques to estimate above-ground biomass of an individual plant. In these, the projected shoot area of the plants captured on two dimensional images was used as a parameter to predict the plant biomass [1,15,21-25]. Except for predicting cereal plant biomass as a linear function of plant area, however, none of the methods described in the literature was developed explicitly for high throughput phenotyping facilities. A robust and accurate method is required for high throughput phenotyping.

An additional factor to consider is the level of salinity to which the plant has been exposed. Arid and semi-arid agricultural lands such as those in Australia inevitably pose some levels of soil salinity, which is one of the major environmental stresses that significantly affects crop productivity. The crop plants are stressed when the high concentrations of salts in the soil make it harder for their roots to extract water [26,27]. Salinity seems to have some effect on wheat growth in terms of their morphology, physiology and anatomical changes [26,28-30]. The applied salt treatment on the plants in the simulates the effect of soil salinity on crop plants in an agricultural field. The linear model, the predominant method used to estimate plant biomass, shows biased estimation of plant biomass particularly for salt stressed plants.

The objective of the present study is to develop a generalized method to estimate the biomass of cereals from their projected shoot area on two dimensional images. We have developed a method that significantly reduces the bias in biomass estimation of stressed cereal plants, which is the main source of the estimation error. We have demonstrated that a model that uses mixed variables of plant area and plant age achieves this reduction and therefore the method we proposed can be used to compute accurately the biomass of cereal plants regardless of whether or not they are salt stressed. In order to generalize our method to cereal plants we tested our method on both wheat and barley datasets and achieved promising results.

## Methods

### Image acquisition

Plant images were captured using a LemnaTec 3D Scanalyzer (LemnaTec, GmbH, Wuerselen, Germany) at Australian Centre for Plant Functional Genomics (University of Adelaide, Waite Campus, Adelaide). Comparable imaging systems are also used in other phenotyping facilities. Three 1280 × 960 resolution RGB images were taken of every plant: one top view image and two side view images at a 90° horizontal rotation. The images were stored in PNG format. In order to increase the accuracy of separating the background from the region of interest (plant region), a roughly uniform blue background was used and the plant pot was also wrapped in a blue paper tube at the time of imaging. To develop the model and ensure sufficient variation, a total of 320 wheat plants were used for this study. The plants were of two Australian bread wheat varieties, Krichauff and Berkut, grown under two salt treatments, 0 and 100 mM NaCl. The bread wheat (*Triticum aestivum L.*) cultivars Berkut and Krichauff are quite distinct, and have different pedigrees. Berkut comes from CIMMYT, Mexico and Krichauff is a southern Australian commercial cultivar. They have been selected as diverse parents of a mapping population, which have been identified to have significant variation in salinity tolerance traits, one of which has even been mapped in a large genetic study, published recently [31].

In terms of salinity, 100 mM salinity is a moderate level of salinity which reduces growth by approximately 10 to 30%. This level of salinity has been estimated to cover as much as 69% of the Australian wheat belt [32] and is a global problem at this and much higher levels [26].

Seeds were placed on the moist paper towels in petri dishes, closed and double wrapped with polythene bags, and kept under room temperature for 5 to 7 days. Seeds were moisturized every second day. To attain uniform growth, fast growing cotyledons were kept in a cold room for 2 days. Once all the plumules and radicles had reached the length of 3 mm and 4 mm respectively the plants were transplanted into the tubes of a supported hydroponics system in a glasshouse. The experiment was conducted in autumn 2008 in both control (0 mM) and saline (100 mM NaCl) conditions. For the salt stressed plants, the salt was added to the hydroponics at the time of fourth leaf emergence (approximately 12 days after germination) in 25 mM increments and the final concentration of 100 mM was reached as four increments in two days [33]. Five harvests were carried out at 15, 26, 34, 40 and 43 days after planting in the petri dishes. At each harvest time 64 randomly selected plants were imaged and then harvested to measure the shoot fresh weight and shoot dry weight [34,35]. This procedure was used in order to provide variations for plant age of plants. The shoot dry weight measurements vary in the range of 0.025-1.67 g with details given in Table 1.

**Table 1:** Details of Shoot Dry Weight measurements (wheat dataset).

Plant age (days after planting)	Control plants (no salt)		Salt stressed plants		Two salt treatments combined	
	
	SDW (ave)	SDW (stdev)	SDW (ave)	SDW (stdev)	SDW (ave)	SDW (stdev)
15	0.03	0.01	0.02	0.01	0.03	0.01
26	0.17	0.04	0.14	0.05	0.15	0.05
34	0.47	0.10	0.28	0.06	0.37	0.13
40	0.98	0.16	0.51	0.08	0.74	0.27
43	1.17	0.22	0.66	0.16	0.91	0.32

**Grand Total**	**0.58**	**0.47**	**0.33**	**0.25**	**0.451**	**0.39**

The SDW values are given in grams.

SDW = shoot dry weight; ave = average; stdev = standard deviation

The methods described in this study were also tested on barley dataset. Seven barley cultivars (Clipper, Sahara, Vlamingh, Buloke, CPI 71284, Barque, Golden Promise) were grown in a greenhouse (June/July 2008) in a supported hydroponics system as described by [36]. At the time of third leave emergence, NaCl was added to the growth solutions of the stress treated plants in 50 mM steps over two days (early morning and late afternoon) to reach a final concentration of 200 mM NaCl. After 20 and 25 days in hydroponics (8 and 13 days after start of salt stress treatment), two side view and one top view images were recorded before shoots were harvested for measuring fresh weight and dry weight. The barley shoot dry weight measurements vary in the range of 0.016-2.25 g with details given in Table 2.

**Table 2:** Details of Shoot Dry Weight measurements (barley dataset).

Plant age (days after transplant)	Control plants		Salt stressed plants		Two salt treatments combined	
	SDW (ave)	SDW (stdev)	SDW (ave)	SDW (stdev)	SDW (ave)	SDW (stdev)
20	0.124	0.040	0.096	0.045	0.110	0.044
25	0.258	0.103	0.158	0.058	0.210	0.097
49			0.763	0.599	0.763	0.599
**Grand Total**	**0.193**	**0.103**	**0.449**	**0.533**	**0.363**	**0.454**

The SDW values are given in grams.

### Image processing algorithm

We used the LemnaTec 3D Image Analyser (LemnaTec GmbH, Wuerselen, Germany) to run image processing algorithms to extract information from the plant RGB images. The plant color images were first converted into the "Hue Saturation Intensity (HSI)" color model in order to increase the contrast between plant region and background region. A threshold was applied on the hue image in order to separate plant area from the background. The segmentation process was accomplished by selecting the pixels with values over the threshold belonging to plant region and rejecting all the other pixels to the background region. The resulting image is a binary or two-level image, using white and black to distinguish the plant and background regions, respectively. The number of pixels inside the plant region was counted in each of the three orthogonal views, converted to mm^2 ^using the appropriate calibration factor, and then summed to give the *projected shoot area*. This is not the actual shoot surface area but the sum of the areas of the image projected in three planes. There are many cases when a mature plant's leaves are overlapping, appearing behind one another in side view images. In these cases, a top view image provides a means of correction of plant area for those overlapping leaves in side view images. The three orthogonal views (two side views from 90 rotational difference) and a top view correct for hidden areas in the other views and give a robust representation of plant area overall.

**Figure 1:**
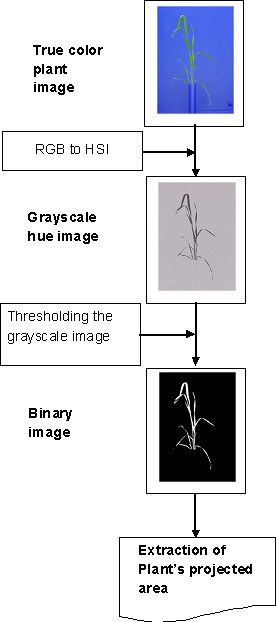
**Process flow of image processing steps used in the extraction of plant's projected shoot area from the images**.

In addition, the top view camera was located at a distance of 2 m above the plant, while plant heights were generally much less than 80 cm. Thus, the camera distance is sufficient for the pixel resolution of leaves near the bottom of the plant to be not too different from that for leaves near the top. Also, the analysis using the two side images alone yields slightly worse results, which demonstrates that the top view is indeed useful. A schematic diagram of the image processing procedure is shown in Figure 1.

### Cross validation technique

To measure the generalization or estimation error of a predictive model, the technique of cross validation was used. Cross validation, or rotation estimation, is a technique for assessing the prediction error. This technique estimates the generalization error, L(Y,Ŷ), where L is the distance function and Ŷ is the model applied to the independent test sample from the distribution of X and Y. Cross validation is a robust method and preferred over the R^2 ^statistic. The main reason is that R^2 ^inevitably increases with additional predictors, and more predictors automatically yield improved prediction within one dataset. However, the cross validation error decreases only as long as the additional predictor improves the predictive capability of the model in an independent dataset [37]. In the cross validation technique, the observations are randomly assigned indices, integers 1 to K. In this way, the dataset is partitioned into K approximately equal-sized parts. Then, the model is fitted to K-1 parts of the dataset (with one part, say the *k*^*th *^part, removed), and the prediction error of the fitted model is calculated from the *k*^*th *^part. This procedure is repeated for *k *= 1, 2, .., K rounds, and estimation errors, such as the root mean square errors (RMSE values), are averaged over the rounds [37-39]. Typical choices of K are 5 or 10, and in this study fivefold (i.e, K = 5) cross validation was used. The estimation errors obtained from applying this technique were used to compare the performance of different predictive models. The cross validation analysis was performed in Matlab (Mathworks Inc., Natick, MA).

## Results and Discussion

### Model development

For our data, the linear method for estimating biomass as a linear function of plant area [14,40] performed better than non-linear models, such as quadratic, cubic and power methods as mentioned in [1,21]. When quadratic and cubic models were fitted, we found that the higher order coefficients were not significant (Table 3). The coefficients are computed from regression modeling using SPSS software package (version 17, IBM, Chicago, Illinois, USA). The models mentioned in this table were developed using the dataset from 320 plants collected in the experiment explained earlier. In these models the plant biomass, or shoot dry weight, was the dependent variable and the projected shoot area was defined to be independent variable. For instance, the linear model is a function with the equation of SDW = a_0 _+ a_1_A, where A is the projected shoot area and SDW is the dependent variable shoot dry weight. The equations associated with the quadratic, cubic and power models are SDW = a_0 _+ a_1_A + a_2_A^2^, SDW = a_0 _+ a_1_A+ a_2_A^2 ^+ a_3_A^3 ^and SDW = a_0 _A^a1^, respectively.

**Table 3:** Significance of regression coefficient of different methods used to estimate plant biomass from the plant area.

Model	Coefficient	Coefficient value	Std. Error	t	**Sig**.
Linear	a_0_[g]	-.043	.008	-5.517	.000
	a_1_[g/mm^2^]	.003	.000	80.412	.000

Quadratic	a_0_[g]	-.046	.011	-3.989	.000
	a_1_[g/mm^2^]	.003	.000	22.237	.000
	a_2_[g/mm^4^]	-1.002E-7	.000	-.347	.729

Cubic	a_0_[g]	-.065	.015	-4.231	.000
	a_1_[g/mm^2^]	.004	.000	11.123	.000
	a_2_[g/mm^4^]	-3.153E-6	.000	-1.911	.057
	a_3_[g/mm^6^]	4.458E-9	.000	1.879	.061

Power	a_0_[log(g)]	4.072E-4	.087	-89.243	.000
	a_1_[log(g)/log(mm^2^)]	1.348	.018	74.655	.000

As can be seen from the Table 3, among polynomial models, only the linear model is significant. The linear model may be compared with the non-linear power model by inspecting their estimation errors achieved using five fold cross validation analysis applied on the wheat dataset.

Table 4 summarizes the root mean square errors (RMSE) from these two models. The RSME is given by:

$$\sqrt{\frac{1}{n}\sum_{k=1}^{n}{\left({\text{SDW}}_{\text{predicted}}-{\text{SDW}}_{\text{actual}}\right)}_{k}^{2}}$$

**Table 4:** Estimation error for linear and power models used to estimate plant biomass.

Plant biomass predictive model	RMSE (g)
SDW = a_0 _+ a_1_A	0.088
SDW = a_0 _A^a1^	0.126

A = Area

where *n *is the total number of images. The estimation error of the linear method is significantly smaller (P-value < 0.00005) than that from the power method (Table 4).

The linear model seems to be the best of those considered so far, justifying its common use in the literature.

**Figure 2:**
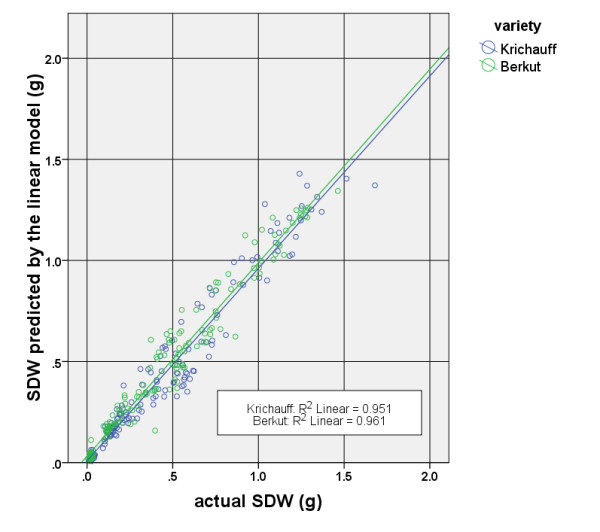
**Scatter plot of the linear method for two wheat varieties.** The blue and green lines are the lines of best fit for two cultivar categories.

When the data were disaggregated into the two bread wheat cultivars, i.e. Krichauff and Berkut, the linear model was still highly significant and could explain greater than 95% of the variance of the values observed. Figure 2 shows the scatter plot of actual SDW values and estimated values obtained by using this method under two variety groups.

**Figure 3:**
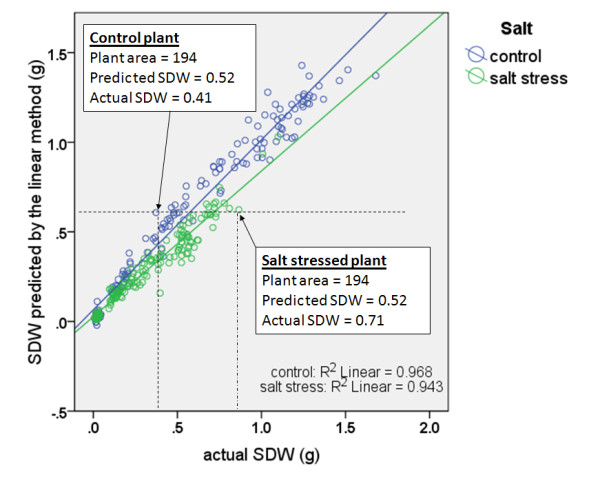
**A scatter plot of actual SDW compared with the estimated values obtained using the linear model for two salt treatment categories**. The blue and green lines are the lines of best fit for two salt treatment categories.

However, this method achieves a large estimation bias for salt-stressed and non-salt-stressed plants. Using this method where the biomass is estimated as a linear function of plant area, the large estimation bias means that plants under salt stress that have the same area (predicted SDW) as the control plants, would in fact weigh more than control plants (greater actual SDW). In other words, this method systematically under-estimated the SDW of salt stressed plants while systematically over-estimating that of plants not under salt stress. An example is illustrated in Figure 3 where example points from two plants under different treatments are highlighted.

**Figure 4:**
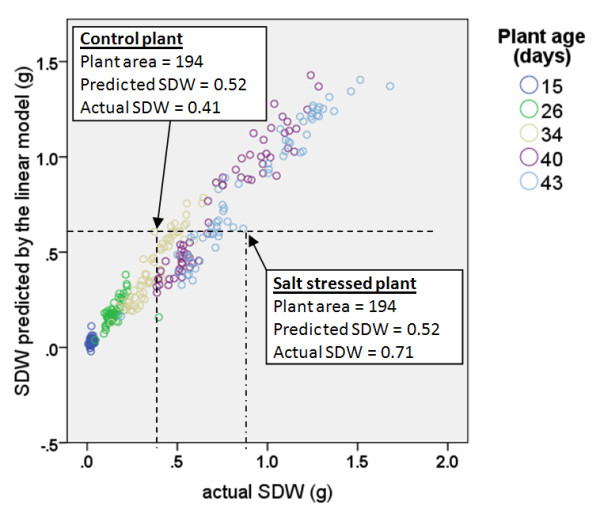
**A scatter plot of actual SDW compared with the estimated values obtained using the linear model for five plant age categories**.

Analysis of the scatter plot of actual SDW values compared with the values estimated by the linear method for five plant ages, however, indicated that plants under salt stress which have the same area (predicted SDW) but greater mass than the control plants are in fact older than those salt free plants (Figure 4). This suggests that the bias observed between the salt-stressed and salt free plants is related to plant age. In this analysis, the plant age is measured from the date of planting.

**Figure 5:**
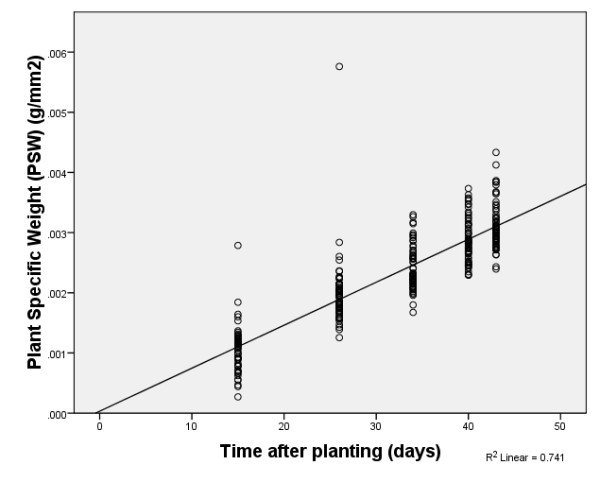
**Scatter plot of Plant Specific Weight in terms of time after planting (plant age)**. A straight line, the line of best fit, seems to describe a plant's PSW in terms of its age.

To reduce the bias, we propose a predictive model based on the concept of plant specific weight. The plant specific weight (PSW) is defined as the plant weight per total projected shoot area. The observations across the images showed that PSW can be estimated as a linear function of plant age (Figure 5). Therefore, PSW could be written as a linear form of plant age, i.e. PSW = b_0 _+ b_1_× plant age.

Our basic model, SDW = Area × PSW, can be extended to:

$$\text{SDW}={\text{a}}_{0}+{\text{a}}_{\text{1}}\times \text{Area}\times \text{density}$$

where SDW means Shoot Dry Weight (g), Area means projected shoot area on the image plane (mm^2^), and the density can be estimated as a linear function of plant age as for PSW above, and a_0 _and a_1 _are the equation coefficients. It is not necessary that the coefficient a_0 _be zero.

Our proposed predictive model can be rewritten as shown in Equation 2.

(2) $$\text{SDW}={\text{c}}_{0}+{\text{c}}_{\text{1}}\times \text{Area}+{\text{c}}_{\text{2}}\times \text{Area}\times \text{HD}$$

where HD is plant age in days after planting.

The coefficients c_0 _to c_2 _were estimated using regression analysis (Table 5). As can be seen from this table, all of these coefficients contribute significantly to the predicted value of shoot dry weight. In our proposed model, the SDW is a function of two inputs of 'Area' and 'Area ×HD' and the coefficients of this model can be computed from a linear regression model, where the values of 'Area' and 'Area × HD' are the independent variables and the values of 'SDW' are entered as the dependant variables.

**Table 5:** Significance of regression coefficient of our proposed method.

Coefficient	Coefficient value	Std. Error	t	**Sig**.
c_0 _[g]	.054	.008	7.229	.000
c_1_[g/mm^2^]	-.001	.000	-5.951	.000
c_2_[g/(day.mm^2^)]	9.866E-5	.000	18.455	.000

### Data analysis and performance comparison of the models

As the linear model proved to be better than the non-linear models we considered, we compared our proposed model with the linear model described in Table 4.

To make it easier to follow, hereafter we refer to the linear model as Model A, and to our proposed model as Model B. The resulting root mean square errors after applying cross validation technique from Model A and Model B are given in Table 6. Model B produces significantly smaller (P-value < 0.00005) RMSE.

**Table 6:** Prediction errors obtained from cross validation method for the linear model and the proposed model.

Predictive model	RMSE (g)
Model A: SDW = a_0 _+ a_1_A	0.088
Model B: SDW = c_0 _+ c_1_A+ c_2_AH	0.058

A = Area, H = Plant age

**Figure 6:**
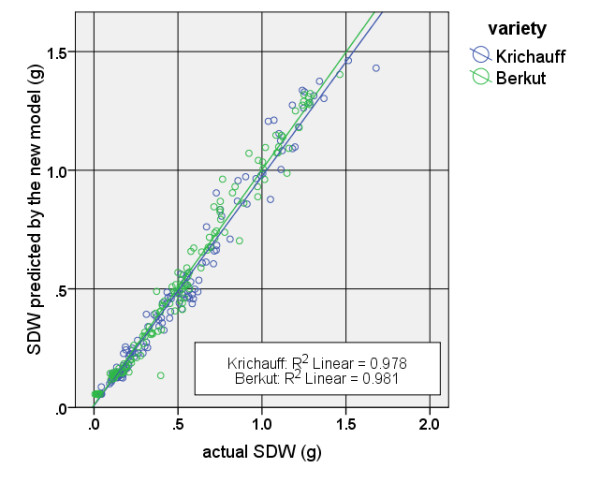
**Scatter plots of actual SDW compared with the estimated values obtained using the model proposed in this study for two wheat varieties**.

**Figure 7:**
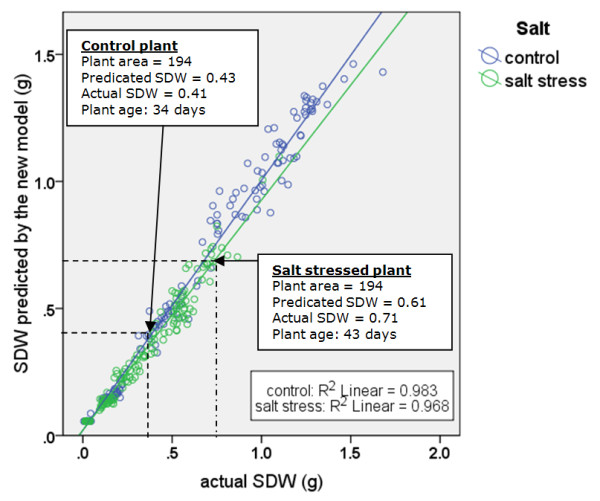
**Scatter plots of actual SDW compared with the estimated values obtained using the model proposed in this study for two salt treatment categories**.

Model B can explain nearly 97% of the dataset observed in two wheat cultivars (Figure 6). Also, it can be seen that by modeling biomass as a function of plant area and plant age, a small estimation bias or difference between actual and predicted shoot dry weight for salt-stressed and non-salt-stressed plants is obtained (Figure 7). By contrast, the conventional approach of modeling biomass solely as a function of plant area (Model A) resulted in larger estimation bias for salt-stressed and non-salt-stressed plants. The two cases highlighted in Figure 3 are highlighted here in this figure as well.

The estimation errors in terms of RMSE and MTE for either of two treatment categories are given in Table 7. The mean total error (MTE), which is referred to as estimation bias, is the average over all images of SDW_predicted _- SDW_actual_. The sign and the magnitude of the MTE indicate whether and how greatly the predicting model under-estimates or over-estimates the SDW value.

**Table 7:** Mean square estimation error and mean total estimation error (bias) for two models under two salt treatment categories.

	Model A		Model B	
**Salt treatment**	**RMSE** **(g)**	**Bias (MTE)** **(g)**	**RMSE** **(g)**	**Bias (MTE)** **(g)**

control	0.090	0.035	0.062	0.011
salt stress	0.076	-0.034	0.052	-0.011

Considering two groups of salt treatments, the root mean square estimation error from Model A is about one and one-half times greater than that for Model B for the control plants and salt-stressed plants. Also, the MTE error for Model A is very high - three times higher than that for Model B for the two groups of salt treatments. According to Table 7, on average the linear model over-estimates the weight of a control plant by 35 mg and under-estimates the weight of a stressed plant by 34 mg. Considering Table 1, this estimation error is about 10% of the mean of all shoot dry weight measurements. Meanwhile, the observations indicate that there is very small bias obtained from Model B to estimate plant biomass. When using the proposed model (Model B), this bias is only 11 mg greater and less than the actual weight of the plant for control and salt stressed plant, respectively.

## Validating the model using barley dataset

Similar observations were achieved by comparing the estimation errors obtained from the linear model (Model A) and the proposed model (Model B) on a series of barley dataset obtained from some selected cultivars. The coefficients of the models were obtained using regression analysis applied on the barley dataset. The models with their regression coefficients and the comparison results in terms of root square of estimation error are given in Table 8.

**Table 8:** Prediction errors obtained from cross validation method on barley dataset for the linear and proposed models.

Predictive model	Coefficients	RMSE (g)
Model A: SDW = a_0 _+ a_1_A	a_0 _= -0.132; a_1_= 0.003;	0.136
Model B: SDW = c_0 _+ c_1_A+ c_2_AH	c_0 _= -0.025; c_1_= 5.48E-4; c_2_= 5.60E-5	0.105

A = Area, H = Plant age

As can be seen, the estimation error for Model B is less than that for Model A. Since the salt application was the main source of estimation bias, RMSE errors obtained from two models for two categories of salt-stressed and non-stressed plants were also compared. The results of this comparison are given in Table 9. We see that Model B achieves a significantly lower RMSE than that for Model A for control plants and RMSE values were approximately the same for the two models for salt-stressed plants. However, the values of MTE errors indicated that on average Model A over-estimated the shoot dry weight of a non-salt-stressed barley plant 93 mg (i.e. 30% of the average shoot dry weight of all plants), which is 10 times greater than the error for Model B. Model B also estimated the shoot dry weight of salt-stressed plants only 5 mg less than the actual shoot dry weight. This bias error was about 10 times less than that for the linear model (Model A).

**Table 9:** Mean square estimation error and mean total estimation error (bias) for two models under two salt treatment categories for barley dataset.

	Model A		Model B	
	**RMSE (g)**	**Bias (MTE) (g)**	**RMSE (g)**	**Bias (MTE) (g)**

control	0.143	0.093	0.032	0.009
salt stress	0.120	-0.047	0.115	-0.005

**Figure 8:**
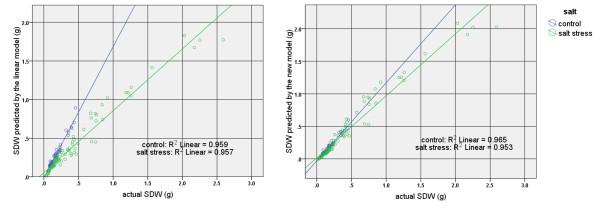
**Scatter plots of actual barley shoot dry weight values compared with the estimated shoot dry weight values obtained from Model A (left) and the Model B (right) under two salt treatment categories**.

Overall, these results confirmed the idea that the plant age, which was used as an additional input for Model B, plays a key role in reducing the error for estimating the plant biomass. This can be seen graphically in the scatter plots of Figure 8 where two regression lines of two salt treatment categories are much closer together and to the line of the best fit for the total values when our proposed method, i.e. Model B, is applied. In contrast, these regression lines are far apart when the Model A is used to estimate plant shoot dry weight.

## Conclusions

In this study we have presented a method for accurate estimation of plant shoot dry weight from two dimensional images. Our proposed model employs information obtained from the images of plants and their age. This approach provides an accurate and practical model for the estimation of wheat and barley shoot dry weight as a substitute for conventional destructive methods of biomass measurement. We also demonstrated that, for salt stressed plants, the estimation bias between the actual and predicted shoot dry weight values can be overcome to a large extent by using plant biomass estimators with plant age as an additional input. Without this method, we cannot accurately infer the plant biomass for salt stressed plants. We tested our proposed model on wheat and barley from different contrasted varieties and under salt stress and found out that with our method the error in biomass estimation was reduced significantly. Thus, our method enables high throughput non-destructive estimation of biomass for cereal plants under salt stress and may possibly do so for other types of plants and stresses.

## Competing interests

The authors declare that they have no competing interests.

## Authors' contributions

KR and BB performed the laboratory work, acquisition and processing of the image data. SR, MT and DSL supervised the study. MRG developed the concept of new model, carried out the data analysis and interpretations, and wrote the manuscript. DSL and RAF contributed to conceiving the study and assisted in data analysis and also in writing the manuscript. All authors contributed in reading, editing and approving the final manuscript.
